# Returning to Yoga Practice and Teaching After Total Hip Arthroplasty

**DOI:** 10.7759/cureus.17669

**Published:** 2021-09-02

**Authors:** Andrew G Yun, Marilena Qutami, Eric Carles

**Affiliations:** 1 Hip and Knee Replacement/Orthopaedic Surgery, Providence Saint John's Health Center, Santa Monica, USA; 2 Hip and Knee Replacement/Orthopaedic Surgery, Providence Saint John's Health Cener, Santa Monica, USA

**Keywords:** total hip replacement, anterior approach, sports, yoga, yoga instructor

## Abstract

Patients who practice yoga are motivated to return to practice after total hip arthroplasty (THA). With case reports of dislocations during yoga, the safety of such a return is unclear. The purpose of this study is to examine the timing and feasibility of a return in a subset of highly experienced and motivated patients. Between 2010 and 2019, a total of 19 THA’s performed in 14 patients who self-identified as yoga instructors were retrospectively reviewed. Patients who practiced yoga but were not teachers were excluded from this series. The primary outcome measures were the ability to return to yoga, to resume teaching, and fluency with 14 classic poses. Secondary outcomes measured were patient-reported Hip Disability and Osteoarthritis Outcome Score (HOOS, Jr.), complications, and radiographic position of the implants. After surgery, all patients returned to practicing and teaching yoga, and the mean time to each was 2 months. All patients were able to perform all 14 classic poses. At a mean follow-up of 5 years (SD ± 4), there were no complications, and the mean HOOS, JR score was 92 points (SD ± 15). This study demonstrates that a return to yoga in an experienced population is not only possible but also safe after a direct anterior THA. Limitations in performing the poses should be understood, and appropriate modifications should be incorporated when needed.

## Introduction

The practice of yoga extends far beyond physical conditioning, with participants seeking a deeper focus on mental, emotional, and spiritual wellness. Its popularity has surged in recent years to include both casual and serious practitioners, and enthusiasm for yoga now extends from the young and healthy to the older and arthritic. The physical practice of yoga incorporates a series of challenging upper and lower body postures to promote strength, balance, and flexibility. Unfortunately, these poses often grow more uncomfortable and difficult with worsening hip arthritis. Weakness, stiffness, and pain necessitate gradual compensatory modifications or even complete cessation. Ultimately, losing the ability to enjoy yoga can be a motivating factor to seek total hip arthroplasty (THA). Whether or not a return to yoga after THA is safe, advisable, or even possible is currently unclear.

A primary concern after THA is dislocation. Conventional post-operative precautions guard against positions of risk, often limiting extremes of hip flexion, extension, adduction, or rotation. However, common yoga poses may place the position of the hip far beyond those boundaries. In a motion capture study of yoga poses in healthy volunteers without THA, Mears et al., noted a mean of over 30 degrees hyperextension in crescent lunge, 109 degrees flexion in pigeon, and 22 degrees rotation in warrior two [1]. Highlighting the risk are three known case reports of hip dislocation during yoga [2,3].

The surgeon’s role, therefore, is to balance the competing demands of a patient’s hope to return to yoga after THA against the risk of dislocation created by the extreme hip positions intrinsic to this discipline. To our knowledge, there are no known studies on yoga after THA; therefore, the purpose of this observational study was to examine the feasibility and safety of resuming yoga after surgery. We characterized the return to yoga practice and the return to yoga teaching in a consecutive series of yoga instructors following THA. We specifically chose instructors because of their domain-specific knowledge after decades of practice, years of instruction, and hundreds of hours of teacher training needed for certification. This proof-of-concept study examines the surgical details, clinical outcomes and limitations, radiographic metrics, and failures.

## Materials and methods

We retrospectively reviewed a total of 19 THAs performed between 2010 and 2019 in 14 patients who self-identified as yoga instructors. Patients who practiced yoga but were not teachers were excluded from this series. Surgery was performed by a single surgeon at a single institution with a minimum of 1-year follow-up. In all patients, a direct anterior (DA) approach THA with intraoperative fluoroscopy was performed. Charts were reviewed for indications, medical history, complications, reoperations, and rehospitalization. IRB approval was obtained from Providence Saint Joseph Health Internal Review Board under STUDY2021000057. Yoga teachers were interviewed for their progression in returning to practice and to teaching following surgery. The primary outcome measures were the ability or inability to perform 14 classic poses [1]. Secondary outcomes measured were patient-reported HOOS, Jr. validated scores and radiographic position of the implants. The study was approved by the Institutional Review Board.

Surgical technique

The patient was placed on the Hana table in the standard fashion. A standard DA approach THA was performed as described by Matta [4]. Standard fixed-bearing components using the largest head size possible were chosen in each case. Intraoperative fluoroscopy with digital radiographic software (Radlink; El Segundo, USA) was used to confirm implant sizing, orientation, and position. Postoperatively, the patients were allowed to proceed with weight-bearing as tolerated and no other precautions. Patients were counseled not to resume yoga for 12 weeks postoperatively.

Radiographic measurement

Postoperative radiographs were evaluated for cup orientation, amount of lengthening, and residual leg length inequality. Anteversion was calculated using the opening of the ellipse as calculated by the Radlink software. Leg length was determined by the vertical distance from a specified point on the lesser trochanter to a horizontal line drawn across the bottom of each radiographic teardrop. Inequality was calculated by the difference in hip lengths between the index side and the contralateral side. The implants were evaluated for loosening, migration, and radiolucent lines as previously well-described [5].

## Results

Of 19 THA cases, 10 cases (53%) were left hips and nine cases (47%) were right hips. Patients included 11 women (79%) and three men (21%); five patients underwent staged bilateral DA THA, and each hip was included as an individual data point. The average age of patients at the time of index surgery was 54 years (range, 35 to 72). The indication for surgery was osteoarthritis (OA) in 17 hips (89%) and avascular necrosis (AVN) in two hips (11%). The demographic information is shown in Table 1.

**Table 1:** Yoga Instructor Cohort Descriptive Statistics including demographic information and pre-operative diagnosis.

Number of cases	19
Number of patients	14
Number of bilateral hip patients	5
Male	1
Female	4
Age (years)	
Mean	54 (SD ± 10)
Range	35 – 72
Gender	
Male	3 (21%)
Female	11 (79%)
Laterality of surgery	
Right	9 (47%)
Left	10 (53%)
Pre-operative diagnosis	
Osteoarthritis	17 (89%)
Avascular necrosis	2 (11%)

At the time of surgery, the average estimated blood loss (EBL) was 234 mL (SD ± 172). For men, the mode acetabular shell size was 58 and the mode head size was 36. For women, the mode acetabular shell size was 50 and the mode head size was 36. There were no intraoperative complications. No patient required an allogeneic transfusion postoperatively. The average length of stay was 1 day (SD ± 1). All patients were discharged to home.

All patients returned to teaching yoga, and the mean time to teaching yoga was 2 months (SD ± 3 months) after surgery. The instructors explained that they could begin teaching class well before needing to participate or demonstrate poses. Financial considerations were described as the main motivation for a timely return to work. Their advice for patients who wish to return to yoga is described in Table 2.

**Table 2:** Instructor Advice for Returning to Yoga Qualitative advice for return to yoga from instructors who have already had a hip replacement.

Let pain be your guide.
Go slowly. Use props.
Blocks help with advanced poses.
Focus on alignment and stability more than depth.
Focus on mind, body connection.
Stop testing for pain.
Don’t do anything that feels weird.
Don’t try anything you were not able to do before surgery.
Adopt a beginner’s mind. Start back in a Level I class.
Start with neutral positions.
Work with a yoga instructor that understands how to return from hip replacement.
Check your ego.
Allow yourself to heal.

**Figure 1:**
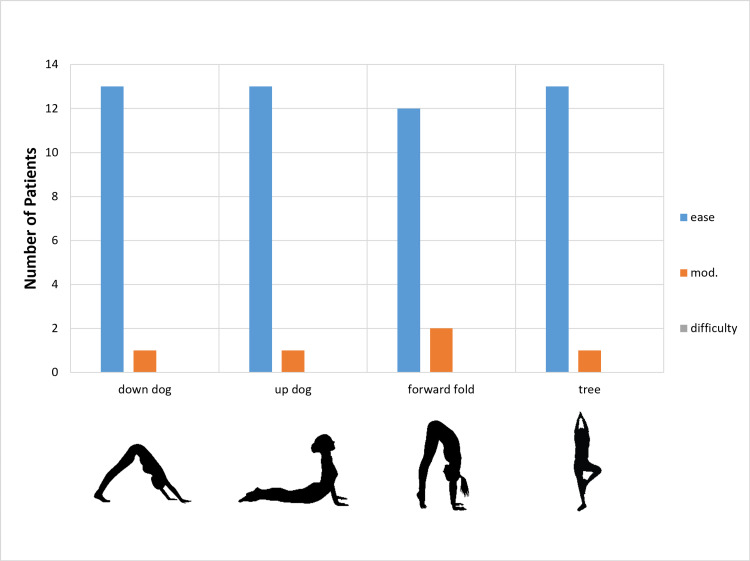
Degree of Difficulty for Specific Yoga Poses Degree of difficulty for return to a specific pose by yoga instructors after total hip replacement.

**Figure 2:**
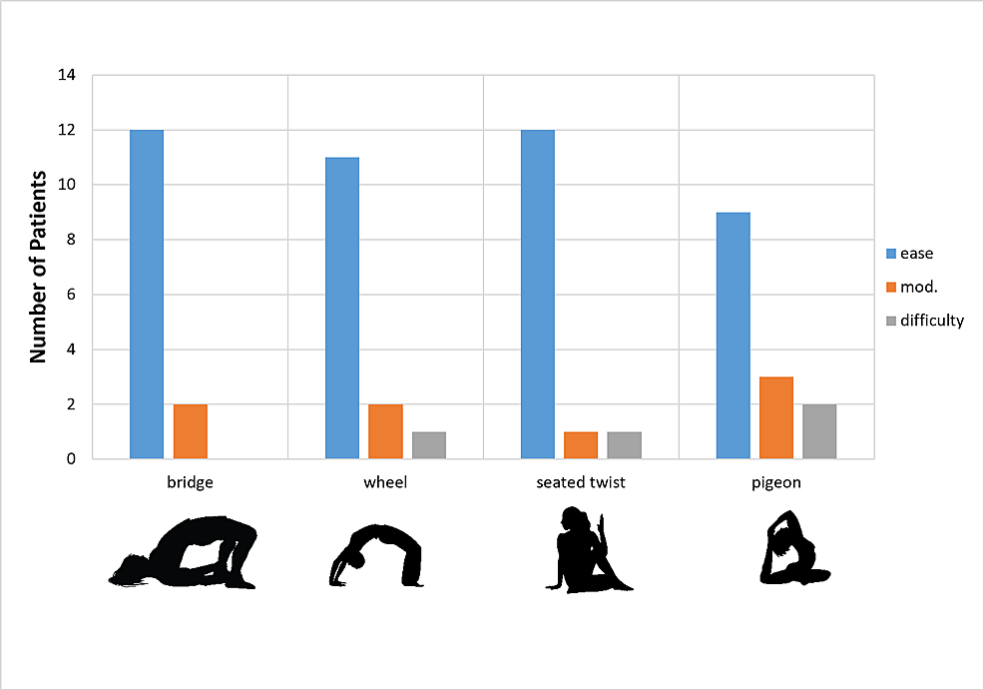
Degree of Difficulty for Specific Yoga Poses Degree of difficulty for return to a specific pose by yoga instructors after total hip replacement.

**Figure 3:**
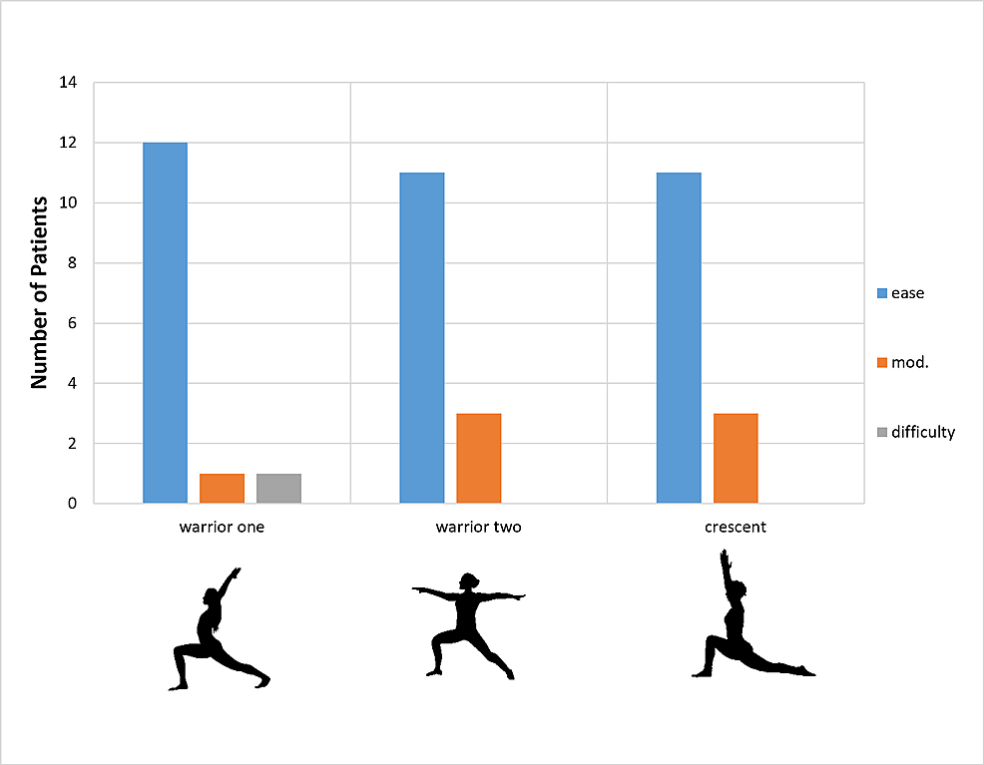
Degree of Difficulty for Specific Yoga Poses Degree of difficulty for return to a specific pose by yoga instructors after total hip replacement.

**Figure 4:**
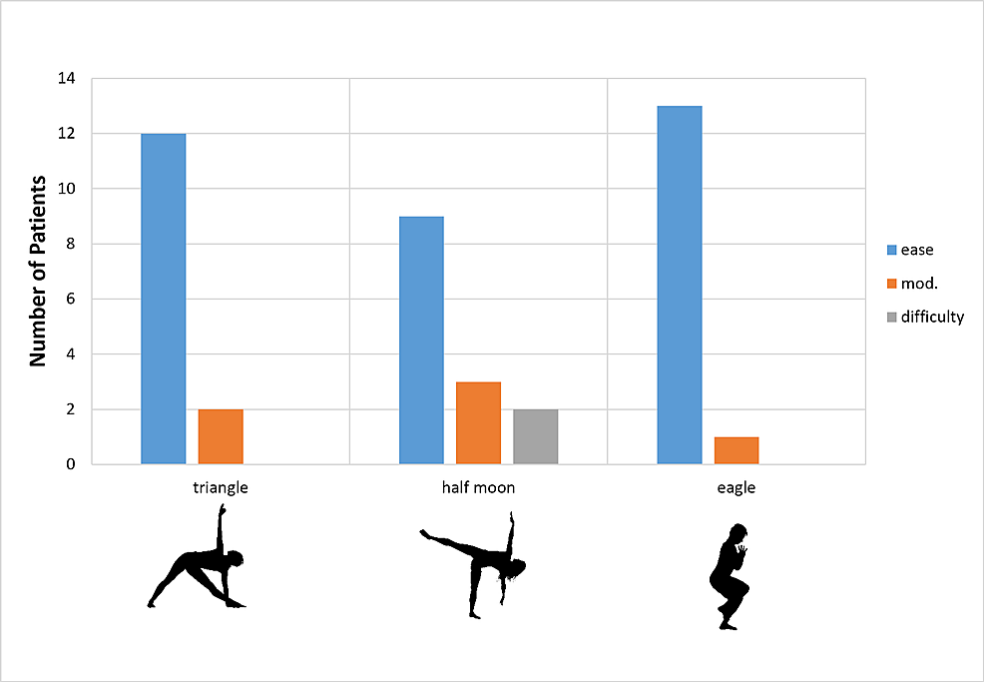
Degree of Difficulty for Specific Yoga Poses Degree of difficulty for return to a specific pose by yoga instructors after total hip replacement.

All patients were able to return to practicing yoga. The mean time to return to yoga practice was 2 months (SD ± 1 month), despite recommendations to the contrary. All patients were able to do all 14 poses with varying levels of fluency. The ability to perform 14 classic poses with ease, with modifications, or with difficulty is described in Figures 1-4. Nine patients (64%) were able to do all poses without difficulty; five patients (36%) reported difficulty with one of four poses (seated twist, pigeon, half moon, and warrior one). Those who reported difficulty with certain poses chose positional adjustments to practice an easier version of the given pose.

Modifications included the need for blocks, straps, and pillows to maintain the classic pose.

At a mean follow-up of 5 years (SD ± 4), the mean HOOS, JR score was 92 (SD ± 15). A preoperative HOOS, Jr score was not available for comparison. Patient self-reported measures can be seen in Table 3.

**Table 3:** Self-Reported Measures Self-reported outcome measures and return to activity. HOOS, Jr score: Hip Disability and Osteoarthritis Outcome Score

Return to yoga practice (months)	
Mean	2 (SD ± 1)
Range	1 - 6
Return to yoga teaching (months)	
Mean	2 (SD ± 3)
Range	1 – 12
HOOS, Jr. score	
Mean	92 (SD ± 15)
Range	65 – 100

With regard to radiographic outcomes, the acetabular implant had a mean abduction angle of 39 degrees (SD ± 3), and a mean anteversion angle of 19 degrees (SD ± 3). The average amount of postoperative limb length discrepancy (LLD) was 2 mm (SD ± 1). At follow-up, all hips showed well-fixed implants. Radiographic findings can be seen in Table 4.

**Table 4:** Radiographic Findings Acetabular component position and leg length measurements after hip replacement.

Abduction (degrees)	
Mean	39 (SD ± 3)
Range	32-45
Anteversion (degrees)	
Mean	19 (SD ± 3)
Range	14 - 24
Leg length discrepancy (mm)	
Mean	2 (SD ± 1)
Range	0 - 4

## Discussion

The question of a return to sports after THA grows more significant with a gradual change in patient demographics and expectations. While studies exist for golf and tennis, there are no known studies examining the return to yoga [6,7]. Current recommendations by surgeons regarding yoga have evolved over the years, and are mostly derived from surveys of surgeons based on personal preferences and community standards [8]. Recommendations to avoid or engage in a particular activity are thus made to patients with only a theoretical or cursory awareness of the risks and possibilities. A 2009 survey of the American Association of Hip and Knee Surgeons regarding a return to athletics did not even address the topic [9]. In a more recent 2020 web-based survey of the European Hip Society, up to 88% of surgeons allowed patients to return to yoga after 6 months without further guidelines [10].

The potential dangers of a return to yoga, however, are well-described anecdotally in three case reports. Tripuraneni et al. described an anterior dislocation 6 weeks after DAA THA successfully treated with closed reduction [3]. Adrados et al. reported two posterior dislocations during yoga [2]. One 43-year-old dislocated posteriorly during a shoulder stand 17 years after index THA, and one 90-year-old dislocated posteriorly 9 years after index THA during a forward fold. All three patients were managed conservatively without further surgery.

Despite informed decision-making regarding these risks, the return to athletics is highly influenced by the patient's motivation to return to the activity after surgery. Many of the patients in this series admitted to a return to yoga well before recommendations. Studies show that a return to a specific sport is only in part determined by the surgeon’s recommendations [11] and largely by a patient’s level of motivation [8]. Boonin et al. identified that 50 to 100% of highly and very highly motivated patients returned to their respective sport regularly after THA [11]. More specifically, up to 80% of highly motivated patients returned to yoga after surgery [8]. In this series, all patients were able to return to their yoga practice and to their role as instructors, and often returned prior to advised timing guidelines.

Surgeons are thus challenged to counsel a patient about the risks of an activity of which they may hold only a limited understanding. A motion analysis study by Mears et al. is an excellent guide that correlates the extremes of motion placed on each hip in 12 common postures [1]. In this series, nearly all patients were able to perform nearly all classic poses without difficulty. Some poses, however, required modifications for safety or comfort. Understanding the degree of flexion, extension, adduction, and rotation placed on the hip in a given pose may inform more actionable recommendations to minimize the risk of instability correlated with a surgical approach.

The role of surgical choices in supporting a patient's return to yoga is unclear. We acknowledge that the decisions to use large heads and a muscle-sparing approach such as the DA are only theoretically based without a control group for the study. We also theorized that eliminating implant position outliers with adjunctive intraoperative fluoroscopy would improve stability, although even well-placed implants are not protective against dislocation [12]. Additionally, we used fixed-bearing constructs, but do not disagree with Acuña et al. who recommended the use of dual mobility bearing in their algorithm for these higher risk patients [13]. Finally, in a retrospective comparison of posterior and anterolateral approaches, Bonnin, et al, found no significant differences in the level of sports participation after 2 years [11]. Future study of optimal bearing and approach in patients wishing to return to yoga is warranted.

There are several limitations of this study. It is a single surgeon’s small retrospective series. Larger numbers of patients and longer follow-up may reveal a more severe complication profile. We chose a minimum one-year follow-up for analysis because our primary endpoints were return to yoga, complications, and implant position. However, longer follow-up may reveal late complications, as dislocations in the case reports were reported many years after surgery. Another major limitation is the lack of a control group for comparison. A cohort of patients treated with a posterior approach and intraoperative imaging could potentially report favorable findings similar to ours. Alternatively, as mentioned previously, a comparative analysis of fixed-bearing versus mobile-bearing designs in yoga patients may clarify the role of implant choice. The purpose of this study, however, was not to argue for a superior approach or technique in these patients; rather, it was to examine the possibility, risks and physical limitations of a return to a challenging sport in a highly motivated and experienced population. An additional limitation is that these results may not translate to yoga practitioners that lack the experience and knowledge of this particular study group. Finally, with the recent transition to patient-reported outcome using the HOOS, Jr. score, a preoperative score was not available for comparison.

## Conclusions

In the absence of definitive evidence-based guidelines, it is clear that motivated patients are likely to resume their yoga practice. It is therefore helpful for surgeons to steer patients toward a safe and gradual return. While the dangers of hip instability inherent to yoga are anecdotally well-documented, the guidelines among arthroplasty surgeons are vague. This study demonstrates that a return to yoga in an experienced population is not only possible but also safe. Limitations in performing the poses should be understood, and appropriate modifications should be recommended when needed. The advice from teachers who have safely and successfully returned to practice should be well-heeded. Future comparative studies in implant choice and approach, as well as a growing understanding of the biomechanics involved, will clearly improve upon current guidelines. Until then, it appears that motivated practitioners may safely return to yoga after a DA total hip replacement.
